# Digitalization-induced changes in health and social care work - perceptions of professionals

**DOI:** 10.1093/eurpub/ckac129.424

**Published:** 2022-10-25

**Authors:** A Kaihlanen, J Nadav, E Kainiemi, T Heponiemi

**Affiliations:** Finnish Institute for Health and Welfare, Helsinki, Finland

## Abstract

**Background:**

Digitalization has increased rapidly in health and social care and plays an increasingly important role in the daily work of health and social care professionals. The effects of digitalization are often viewed from the societal and economic perspectives, and less from the perspective of the changing health and social care work. This study examined how health and social care professionals perceive the effects of digitalization on their work.

**Methods:**

Eight semi-structured focus-group interviews were conducted in four Finnish health centers at the end of 2020. The participants (n = 30) were nurses, physicians, and social workers. Qualitative content analysis with inductive approach was used to analyze the data.

**Results:**

Four main categories emerged from the perceived effects of digitalization: 1) two-way changes in workload and pace (reduced/increased work, accelerated/slowed pace of work, duplication of work/saved employee resources), 2) changes in the content and nature of work (reallocation of work, emergence of new tasks, new skills needs, diversification of service provision), 3) changes in work community communication and interaction (improved interaction and communication, strengthened multidisciplinary collaboration, complicated remote interaction, reduced encounters) and 4) improved flow of patient information and information security (improved data transfer, patient monitoring and data protection).

**Conclusions:**

Digitalization-induced changes in health and social care work seem to be manifold and often two-sided. It has the potential to ease the work and offer other benefits, but at the same time it may complicate work in other respects, especially if the system does not support work tasks or the usability is poor. When implementing new digital services and pursuing benefits, more attention should be paid to assessing and considering the potential disadvantages to minimize additional strain among already burdened health and social care professionals.

**Key messages:**

• The perceived effects of digitalization on health and social care work are often two-sided and can contribute to the well-being of professionals.

• Monitoring the use of digital services and the experiences of professionals about them, as well as identifying their skills needs and training aspirations is crucial.

